# Neural crest development and disorders: from patient to model system and back again – the NEUcrest conference

**DOI:** 10.1242/bio.060530

**Published:** 2024-06-17

**Authors:** Marco Antonaci, Amy Kerr, Merin Lawrence, Francesca Lorenzini, Nitin Narwade, Chloé Paka, Anna Magdalena Wulf

**Affiliations:** ^1^ School of Biological Sciences, University of East Anglia, Norwich Research Park, Norwich, NR7 7TJ, UK; ^2^ School of Biological and Chemical Sciences, University of Galway, Biomedical Sciences Building, Second Floor North, Newcastle Road, Galway, H91 W2TY, Ireland; ^3^ Experimental Cancer Biology Laboratory, CIBIO, University of Trento, Trento, Italy; ^4^ Cell plasticity in development and disease Unit, Instituto de Neurociencias, CSIC-UMH, Sant Joan de Alicante, 03550 Alicante, Spain; ^5^ STEMCELL Technologies UK Ltd, Cambridge, UK; ^6^ Centre for Craniofacial and Regenerative Biology, King's College London, London, UK

**Keywords:** Neural crest (NC), Neurocristopathies (NCPs), Development, Migration, Epithelial to mesenchymal transition (EMT)

## Abstract

The neural crest (NC) is an embryonic multipotent and transitory population of cells that appears during late gastrulation/early neurulation in the developing embryos of vertebrate organisms. Often called “the fourth germ layer”, the NC is characterised by incredible mobility, which allows the NC cells to migrate throughout the whole embryo, giving rise to an astonishing number of different derivatives in the adult organism, such as craniofacial skeleton, adrenal gland, enteric nervous system and melanocytes. Because of these properties, neurocristopathies (NCPs), which is the term used to classify genetic diseases associated with NC developmental defects, are often syndromic and, taken all together, are the most common type of genetic disease. The NEUcrest consortium is an EU funded innovative training network (ITN) that aims to study the NC and NCPs. In March 2024, the early stage researchers (ESRs) in the NEUcrest consortium organised an in-person conference for well-established and early career researchers to discuss new advances in the NC and NCPs field, starting from the induction of the NC, and then moving on to migration and differentiation processes they undergo. The conference focused heavily on NCPs associated with each of these steps. The conference also included events, such as a round table to discuss the future of the NC research, plus a talk by a person living with an NCP. This 3-day conference aimed to bring together the past, present and future of this field to try and unravel the mysteries of this unique cell population.

## Teaser

**Summary:** In March 2024, the early-stage researchers in the NEUcrest consortium organised an in-person conference for well-established and early career researchers to discuss new advances in the neural crest and neurocristopathies field.

## Introduction

**Fig. 1.**
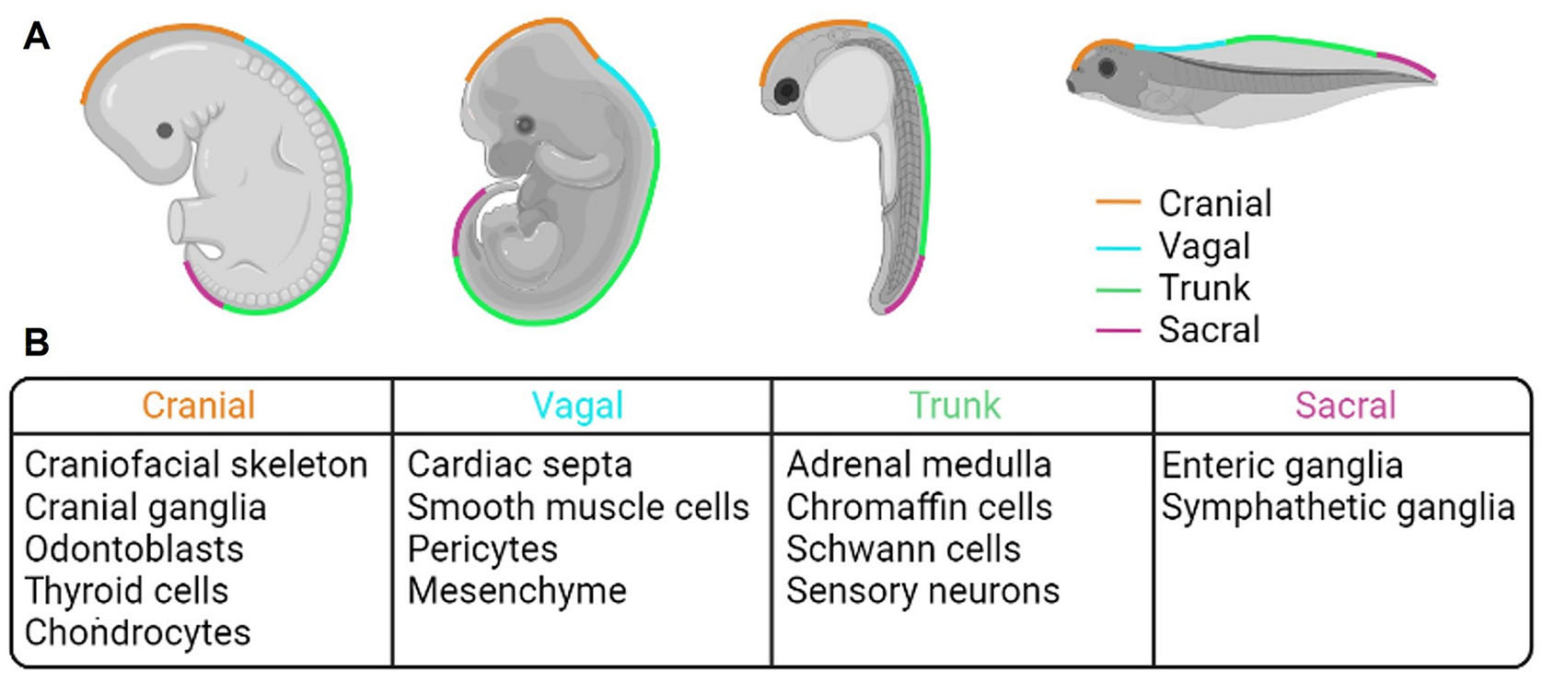
**Neural crest contribution in the adult organism.** (A) NC separation in different animal models during embryogenesis (from left to right: human, mouse, zebrafish and frog). (B) Examples of NC contribution to the adult organism, specific for the different NC populations. All the NC populations give rise to melanocytes in the adult organism.

The neural crest (NC) is a transient cell type that originates during late gastrulation/early neurulation of a developing embryo. During development, neural crest cells (NCCs) are induced at the neural plate border, and then undergo a process called epithelial to mesenchymal transition (EMT), which allows them to migrate throughout the embryo. These cells then differentiate into an astonishing number of derivatives, which include (but are not limited to) craniofacial skeleton, teeth, melanocytes, enteric ganglia, and adrenal medulla (Fig. 1) (Bronner and LeDouarin, 2012). Such an incredible cell population is necessarily controlled by very complex gene regulatory networks (GRNs), which are constantly being updated with the addition of new transcription factors, signalling molecules and non-coding RNAs (Simoes-Costa and Bronner, 2013, 2015; Seal and Monsoro-Burq, 2020; Pla and Monsoro-Burq, 2018; Antonaci and Wheeler, 2022).

Diseases associated with problems during NC development are often syndromic, like Waardenburg syndrome (Huang et al., 2022), CHARGE syndrome (Usman and Sur, 2021), or DiGeorge syndrome (Lackey and Muzio, 2021). However, if the problem occurs later during NC development, it can give rise to NCPs with very specific phenotypes, such as Hirschsprung disease (Lotfollahzadeh et al., 2021), craniosynostosis (Goos and Mathijssen, 2019) or even give rise to some NC-specific cancer types, such as neuroblastoma (Wulf et al., 2021), pheochromocytoma (Gimenez-Roqueplo et al., 2023) and melanoma (Castro-Perez et al., 2023). Given this incredible heterogeneity of disorders, NCPs are, collectively, the most prevalent type of genetic diseases (Vega-Lopez et al., 2018).

Because of the high medical importance, and intriguing nature of the NCCs, in 2019 Professors Anne-Helene Monsoro-Burq, Karen Liu, Grant Wheeler and Gerhard Schlosser organised an innovative training network (ITN), funded by the EU program Horizon2020, NEUcrest – Training European Experts in Multiscale Studies on Neural Crest Development and Disorders: from Patient to Model System and Back again.

**Fig. 2.**
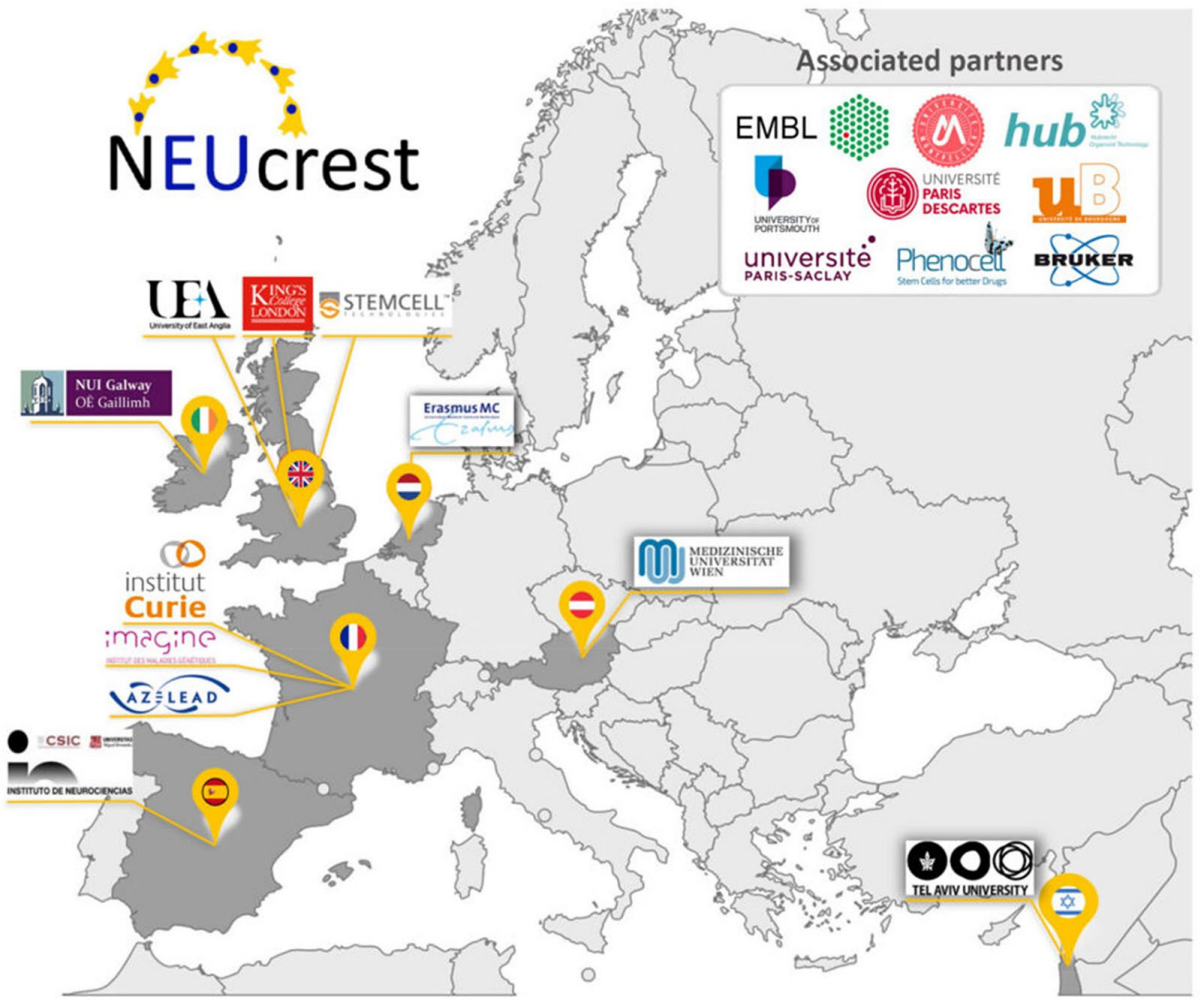
**Map of the NEUcrest partners across the European territory.** Map showing all the partners (academic and industry) that are involved in the NEUcrest ITN. The Countries in dark grey are the ones hosting the early stage researchers (ESRs).

With a total of 11 beneficiaries, and nine associated partners (both in academia and industry), NEUcrest has the objective of training 15 early stage researchers (ESRs) and exploring different aspects of NC biology, from its physiology to pathological conditions (https://neucrest.curie.fr/index) (Fig. 2).

The conference ‘Neural crest development and disorders: from patient to model system and back again’, held in the peninsula of Giens (France), at the Belambra Club, between the 3rd and the 6th of March 2024, celebrated the culmination of this program. Organised by selected members of the ESRs with help from the PIs, this in-person conference aimed to bring together well-established experts with scientists just starting their careers in this exciting field. Alongside talks from many invited speakers, this conference, gave the ESRs in the consortium the chance to present the results that they had obtained during the course of their PhD projects within the NEUcrest.

In this Meeting Report, we highlight the exciting content of this friendly conference of 100 people, in which established scientists from across the globe reunited with old friends, and young researchers had the chance of engaging for the first time with fellow scientists that they will, hopefully collaborate with in the future.

## From induction to differentiation: a neural crest trip

As a conference opener and keynote speaker we had the honour of hosting Professor Marianne Bronner (Caltech, USA). She and her lab are one of the leading forces in to NC development in all of its facets (Martik and Bronner, 2021; Solovieva and Bronner, 2021; Tang and Bronner, 2020). Her presentation introduced the inspiring work that her lab is carrying out on the contribution of the NCCs to cardiac development. Using a variety of techniques, in zebrafish and chick embryos, they have found that *Tgif1* and its downstream targets are required to induce the cardiac identity of NCCs.

The way the NEUcrest conference was organised aimed to transport the attendees on a journey, following the developmental steps of the NCCs. Because of this, the early sessions were mainly focused on the induction and specification of the NC and as the conference progressed the talks shifted more towards differentiation of the NC.

One important aspect that emerged during the conference was that no matter the stage of NC development that the research group is investigating, a great importance is given to the medical impact that the research can lead to. The talk from Dr Anestis Tsakiridis (University of Sheffield, UK), was a perfect example of this. His lab is interested in the use of hiPSCs-induced NC with the aim of modelling human diseases, and to potentially use these reprogrammed cells to perform cell replacement therapy for people affected by Hirschsprung disease (Cooper et al., 2024). This neurocristopathy affects the enteric nervous system, generating several symptoms that range from severe constipation to malnutrition.

Historically speaking, developmental biology has focused much on transcription factors, whose expression and regulatory networks have been extensively studied for decades. Because of this, it was good to see that many of the talks were also diversified, pointing at different aspects of cell biology. Dr Eric Theveneau (Centre for Integrative Biology, France), one of the invited speakers, talked about the role that metalloproteases (and MMP14 in particular) play in NC delamination and migration (Barriga and Theveneau, 2020). Another example was given by Dr Ruth Palmer (University of Gothenburg, Sweden) and Anna Wulf (King's College London, UK), who both gave an insight into ALK (anaplastic lymphoma kinase) biology. ALK is the major player involved in the onset of neuroblastoma, a cancer of the sympatho-adrenal lineage which mostly affects children aged 5, or younger. While Palmer uses the *Drosophila* model to study pathological mutations in the *ALK* gene and its involvement in the DNA-damage response (Borenas et al., 2024), Wulf focused on its interaction with GSK3 (glycogen synthase kinase 3), and how the migration of NCCs is affected by pathological mutations of *ALK* (Wulf et al., 2021). The talk from another ESR, Marco Antonaci (University of East Anglia, UK), presented his data that show the dual role of *miR-204-1-5p* and *miR-204-1-3p* during the development of the eye (Antonaci and Wheeler, 2022). Also with the aim of studying human conditions, Dr Ruth Williams (University of Manchester, UK) talked about the role of the chromatin remodelling factor *Chd7*, the causative gene of CHARGE syndrome, using chicken embryos.

As Next Generation Sequencing progresses and becomes more and more accessible, the number of labs that are generating and processing this kind of data keeps increasing. The merging of different datasets produced by RNA, ATAC, ChIP, and Hi-C sequencing has the potential to drawing a clearer image of the molecular mechanisms that drive embryo development. A useful tool has been developed by Nitin Narwade, bioinformatician in Angela Nieto's lab (Institute of Neuroscience, Spain), which is capable of predicting cell fate trajectories in case of perturbation (introduction or removal) of a transcription factor from a still-undifferentiated cell type.

Speaking about cutting-edge technologies, it is impossible not to mention the contribution to the field (and to this conference) given by Professor Igor Adameyko. With his lab's single-cell OMICS, fate mapping and lineage tracing, he is currently one of the leading scientists when it comes to cell fate determination. Several people from his laboratories, located at the Medical University of Vienna (Austria) and at the Karolinska Institute (Sweden), presented their innovative data. One of the invited speakers, Dr Maria Elena Kastriti, is part of his team in Vienna. She presented data from one of her recent publications about the finding of a “hub state” of the Schwann cell precursors, that allows these cells to differentiate into a variety of cell type during mouse embryogenesis (Kastriti et al., 2022). Following that cell lineage, but looking at frog embryos, Amy Kerr (University of East Anglia, UK) presented her work on the characterisation of the development of the adrenal gland. She showed that chromaffin-like cells start appearing very early during development, and hinted that NCCs might be able to migrate even further than originally thought.

Also from the Adameyko lab, another ESR, Irina Poverennaya, introduced her bioinformatical analysis that highlighted the importance of ribosomal RNA and ribosomal assembly transcripts in NC fate decision. Next, Merin Lawrence (University of Galway, Ireland) presented her CUT&RUN data integrated with RNA-seq, aimed to shed light on the role of *SOX10* in placode and NC development.

## Ethical science, stem cells and animal models: the Yin and Yang of research

In the past years, the advent of stem cells and iPSCs technology has revolutionised the entirety of biological science. This is particularly true for the field of developmental biology. The possibility to produce, *in vitro*, NC-induced pluripotent stem cells (NC-iPCs) has encouraged an increasing number of scientists to use less animals in their experiments. Of course, researchers are aware that the use of NC-iPCs is not completely accurate in the recapitulation of the biological context of the NC development in a living organism. It is undeniable, though, that this model has the potential of reducing (but not completely replacing) the number of animals used in science every year.

In a society where the general opinion towards science is torn between the use or not of animals in science, it is important to make an effort and move towards this direction, whenever possible.

For this reason, an entire session of the NEUcrest conference was dedicated to research that uses biological models alternative to animals. During the session called ‘Exploring neural crest biology with 3Rs strategies’, speakers explained their research which gives particular importance to the 3Rs principles of *Replacement*, *Reduction* and *Refinement* of animals in research.

As an example of replacement, one of the ESRs, Ana Filipa Duarte (Erasmus Medical Centre, Rotterdam, Netherlands), described the generation of hiPSCs derived from a person affected by craniosynostosis. This person carries a potentially pathological variant on *FUZ* gene (*FUZ^R284P^*), which she reverted using CRISPR-prime editing, before further differentiation into the osteogenic lineage (Barrell et al., 2022).

Filipa was not the only NEUcrest member working on hiPSCs. Chloe Paka (Stemcell Technologies, UK) and William Bertani-Torres (Imagine Institute, France) are also modelling human neurocristopathies *in vitro*. Chloe, who works closely with Karen Liu's research team, has developed a cell line harbouring a mutation in *ALK* gene, that recapitulates a human mutation that has been found in patients with neuroblastoma. In a similar fashion, William is working on the characterization of a novel human mutation in *ADAMTS20* that has been found in a patient affected by Waardenburg syndrome (Bertani-Torres et al., 2023).

Similarly, Ayat Ahmed (a PhD student at the University of Heidelberg, Germany), also described work using hiPSCs-derived NC to generate cortical neurons or neural progenitor cells. Her aim was to investigate the role of the transcription factor *NR2F1* (‘A gene to rule them all’) and its long non-coding RNA (*lncNR2F1*) in chromatin organisation. By using high throughput techniques, such as Hi-C, 4C-seq, ATAC-seq and ChIP-seq, her goal is to shed light into the etiopathogenesis of the Bosh-Boonstra-Schaaf Optic Atrophy Syndrome (BBSOAS). This rare syndrome is caused by deletions or mutations in *NR2F1* locus, and causes typical craniofacial dysmorphism, autism spectrum disorder, and optic nerve atrophy.

Melanoma is a major cancer in Western society. Francesca Lorenzini (Azelead, Montpellier, France), presented beautiful images generated during her PhD, using zebrafish embryos to study the microenvironment of melanomas by using patient-derived xenograft imaging (Marines et al., 2023).

## The urge for more cooperation between scientists and people affected by NCPs

A particularly special moment of the conference was the presentation of Charlotte Ashby, which was followed by a small discussion round. Charlotte lives with unicoronal Craniosynostosis and was invited to represent people living with these kinds of conditions. She talked about her experience, how it is to go through endless rounds of surgeries and medical discussions and also introduced the foundation she is part of: Headlines Craniofacial Support UK (https://www.headlines.org.uk/). Charlotte is a sociology graduate from the University of Bath and became the youngest-ever trustee of Headlines, at the age of 19.

Charlotte first talked about the troubles her parents encountered when she was a baby, regarding the initial struggle to find a diagnosis, and her parents having to watch their young child go through various medical procedures right after birth. Something that struck us in particular, was Charlottes admittance, that she didn't quite have a good understanding of her own diagnosis when she started being in charge of her own medical treatment courses as a young adult. It made all of us smile, but also reflect, to hear Charlotte opening's sentence: “I have to admit, before being invited at this conference, I had no idea what the neural crest was”. It highlighted an old and deep-rooted problem between scientists and the general public, even with respect to those closely affected by scientific research.

Her knowledge of the condition was mostly based on her own reading and basic explanations of doctors but never went into great detail. She also told us that she was fascinated listening to some of our speakers, talks that were focused on background research on craniosynostosis and their work, and how much work is already being done.

Charlotte then introduced us to the work of Headlines. This charity was initially funded by a group of parents of children with craniosynostosis in 1993. It is currently run mostly by those living with craniosynostosis. Headlines is focused on providing support for people living with craniofacial conditions, raising awareness, educating people to improve public understanding of such conditions and facilitating research on craniosynostosis and other rare craniofacial conditions.

After the talk, there followed a question and discussion round with Charlotte, and the overall consensus seemed to be a wish on both sides to be more involved with each other. Researchers were keen to meet patients and to be able to explain their work. On the other hand, Headlines is interested in raising more awareness of the patient experience but also having more contact and events with researchers to create a greater understanding of such conditions.

Someone asked how many people in the room had worked with patients in any form before, and about 20% of the attendees raised their hand. Overall, this was a unique experience for both Charlotte and for all researchers in attendance and it added depth and meaning to the work presented at the conference. We are very grateful to Charlotte for bravely talking to a room full of scientists for the first time and allowing us to hear her story and about Headlines' work.

## Attendees at the conference: statistics

With its 95 participants, “Neural crest development and disorders: from patient to model system and back again” was a medium-small sized conference that aimed at creating a sense of community among the participants.

Using a combination of short talks and flash talks, 46 of the participants were given the opportunity to give an oral presentation. Also, with a total of 54 posters and eight invited speakers, more than half of the participants to the conference had the chance to present their work (either orally, or with a poster presentation). Of these oral presentations and posters, 12 were provided by the NEUcrest ESRs. This means that 60% of the participants had the chance to present their work, or the work conducted in their lab, through an oral or poster presentation (or both). Of these fractions, the NEUcrest ESRs accounted for 26% of the oral presentations, and 21% of the poster presentations (Fig. 4A). About the country of origin of the attendees, the biggest fraction of them was based in France, as expected (a total of 27 people), 58 people came from a European Country (other than France), and 10 attendees joined from extra European countries (USA and the Republic of China) (Fig. 4B).

**Fig. 3.**
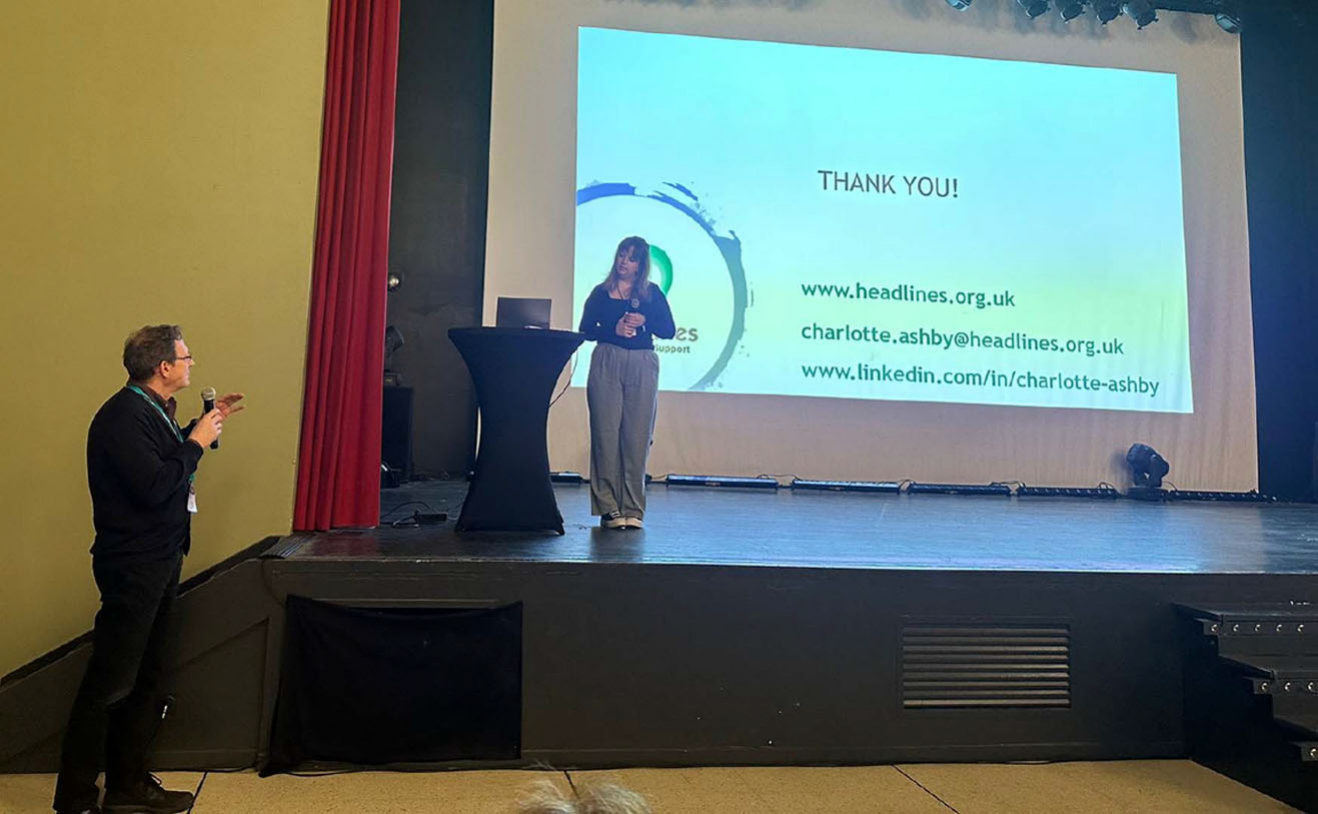
**Picture of Charlotte Ashby's talk, chaired by Professor Grant Wheeler (University of East Anglia, UK)**. Charlotte Ashby was born with unicoronal craniosynostosis and became Headlines' youngest-ever trustee in 2020, at the age of 19. She runs the charity's Young Person's Network, which allows people aged 16-30 with craniofacial conditions to connect. Charlotte is passionate about advocating for young people and adults with craniofacial conditions and utilizing her experiences of growing up and living with a craniosynostosis to raise awareness of the condition.

**Fig. 4.**
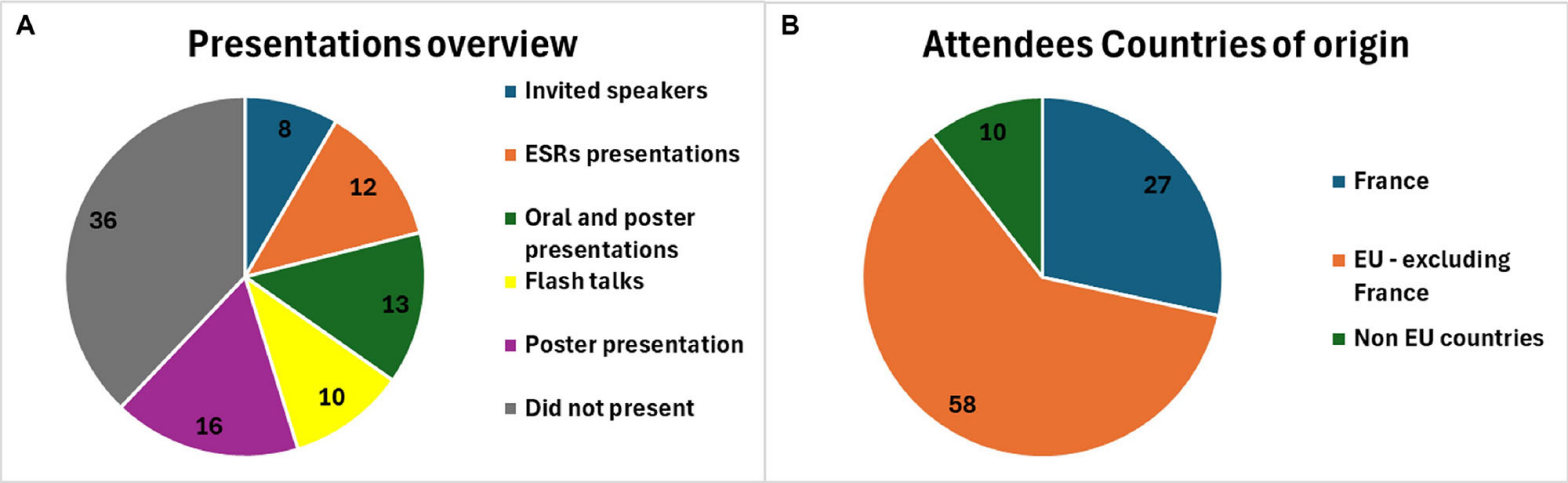
**Attendees at the “*Neural Crest Development and Disorders: From Patient to Model System and Back Again*” conference.** (A) Breakdown of the number of attendees who had the chance of presenting their work through an oral presentation. Over half of the attendees had the possibility to present with an oral or poster presentation, or both. (B) Country of origin of the attendees. Most of them joined from a European country (58/95), with a prevalence of France institutions (27/95). In green, the number of attendees from extra European Countries (mainly USA and Republic of China).

**Fig. 5.**
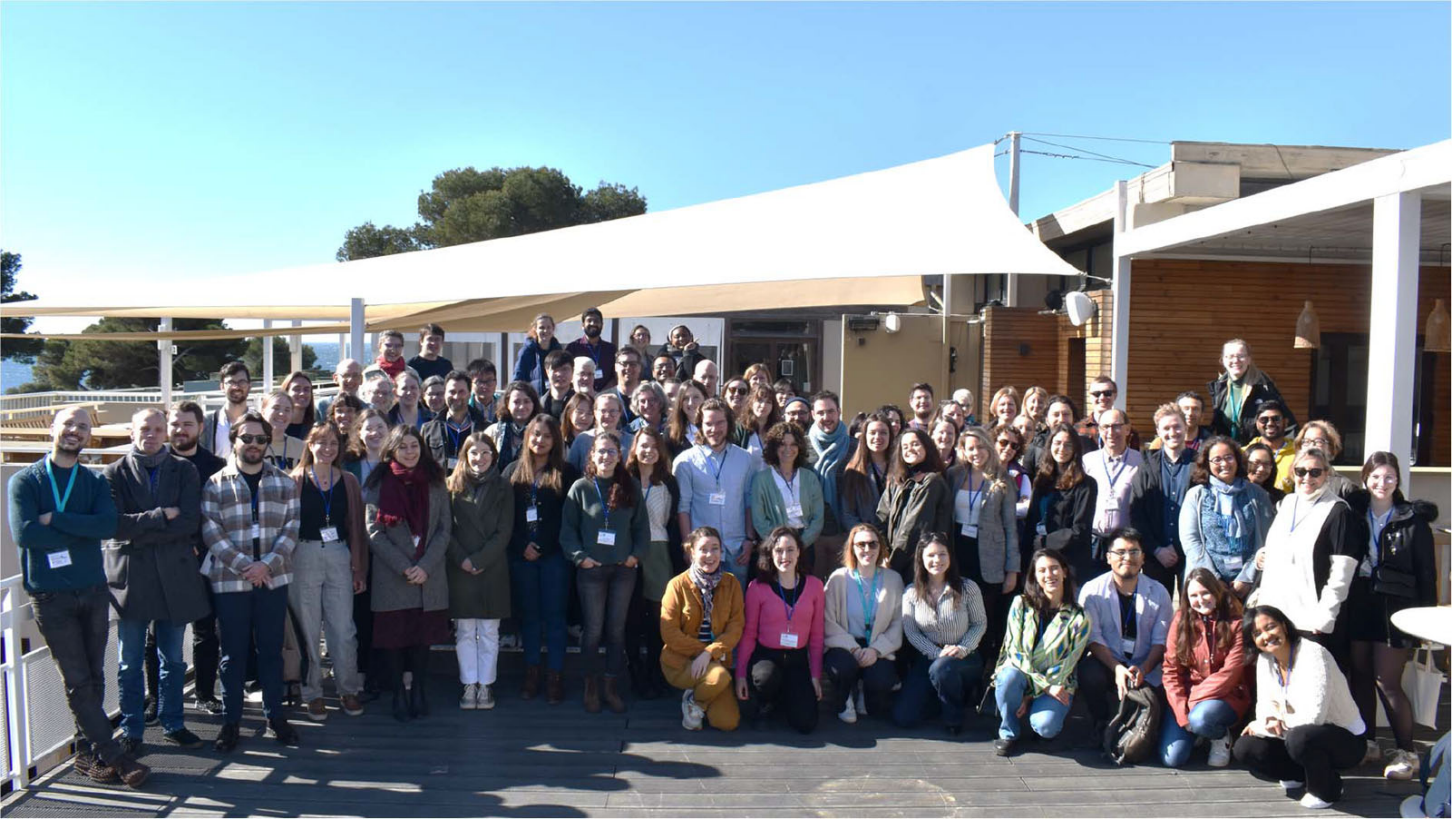
Group picture of the attendees at the ‘Neural crest development and disorders: from patient to model system and back again” conference.

Overall, given the length of the conference (2.5 days in total), it was expected that not so many people would have joined from countries other than European ones. However, people gave positive feedback to the organisers about the number of participants, and said that they had the chance of talking with most of the other attendees during the breaks, or the evenings.

## Conclusion

In March 2024, the conference: ‘Neural crest development and disorders: from patient to model system and back again’ was organised as final milestone and a celebration of the NEUcrest project. This conference, mainly organised by the early career researchers in the NEUcrest consortium, had different goals: to give the chance to young researchers to share their work to a community of experts in the field, and to interact with each other; to bridge the gap between scientist and people affected by rare conditions; discuss the evolution of the NC and, more broadly, the developmental biology field; lastly, to share with the scientific community the goals and achievements obtained by the NEUcrest consortium.

With discussions, sharing of ideas, point of views and vibrant debates, we believe these goals were achieved. Almost 100 participants from across the globe joined the conference. Most of them had the chance to share their results and ideas, either during the poster sessions or during the oral presentations, kept short specifically to allow most people to give a talk, which included some excellent flash talks of 2-3 min each. Unfortunately, in this report, we have not been able to mention every talk from the attendees. Developmental Dynamics had kindly offered prizes of 250$ each for the best talk and poster. These were awarded at the end of the conference to Dennis Pang (University of Hong Kong) for his poster titled: “Elucidating the mechanistic role of STRAP in regulating SNAI2 stability for proper onset of cranial neural crest epithelial to mesenchymal transition” and Sydney Arlis (University of Iowa) for her talk titled: “Defining the role of ISM1 in craniofacial development”.

While the future cannot be foreseen, after this conference we can at least predict the direction that this exciting field is taking: a more ethical science, with an increased level of resolution, but without forgetting clever experimental design and the reasons why we are asking our questions. Either way, with the exciting science that the attendees showed during the conference, we can be quite sure that the future of the neural crest research is in good hands.

## References

[BIO060530C1] Antonaci, M. and Wheeler, G. N. (2022). MicroRNAs in neural crest development and neurocristopathies. *Biochem. Soc. Trans.* 50, 965-974. 10.1042/BST20210828 35383827 PMC9162459

[BIO060530C2] Barrell, W. B., Adel Al-Lami, H., Goos, J. A. C., Swagemakers, S. M. A., Van Dooren, M., Torban, E., Van Der Spek, P. J., Mathijssen, I. M. J. and Liu, K. J. (2022). Identification of a novel variant of the ciliopathic gene FUZZY associated with craniosynostosis. *Eur. J. Hum. Genet.* 30, 282-290. 10.1038/s41431-021-00988-6 34719684 PMC8904458

[BIO060530C3] Barriga, E. H. and Theveneau, E. (2020). In vivo neural crest cell migration is controlled by "Mixotaxis". *Front. Physiol.* 11, 586432. 10.3389/fphys.2020.586432 33324240 PMC7723832

[BIO060530C4] Bertani-Torres, W., Lezirovitz, K., Alencar-Coutinho, D., Pardono, E., Da Costa, S. S., Antunes, L. D. N., De Oliveira, J., Otto, P. A., Pingault, V. and Mingroni-Netto, R. C. (2023). Waardenburg syndrome: the contribution of next-generation sequencing to the identification of novel causative variants. *Audiol Res.* 14, 9-25. 10.3390/audiolres14010002 38391765 PMC10886116

[BIO060530C5] Borenas, M., Umapathy, G., Lind, D. E., Lai, W. Y., Guan, J., Johansson, J., Jennische, E., Schmidt, A., Kurhe, Y., Gabre, J. L. et al. (2024). ALK signaling primes the DNA damage response sensitizing ALK-driven neuroblastoma to therapeutic ATR inhibition. *Proc. Natl. Acad. Sci. USA* 121, e2315242121. 10.1073/pnas.2315242121 38154064 PMC10769851

[BIO060530C6] Bronner, M. E. and Ledouarin, N. M. (2012). Development and evolution of the neural crest: an overview. *Dev. Biol.* 366, 2-9. 10.1016/j.ydbio.2011.12.042 22230617 PMC3351559

[BIO060530C7] Castro-Perez, E., Singh, M., Sadangi, S., Mela-Sanchez, C. and Setaluri, V. (2023). Connecting the dots: Melanoma cell of origin, tumor cell plasticity, trans-differentiation, and drug resistance. *Pigment Cell Melanoma Res.* 36, 330-347. 10.1111/pcmr.13092 37132530 PMC10524512

[BIO060530C8] Cooper, F., Souilhol, C., Haston, S., Gray, S., Boswell, K., Gogolou, A., Frith, T. J. R., Stavish, D., James, B. M., Bose, D. et al. (2024). Notch signalling influences cell fate decisions and HOX gene induction in axial progenitors. *Development* 151, dev202098. 10.1242/dev.202098 38223992 PMC10911136

[BIO060530C9] Gimenez-Roqueplo, A. P., Robledo, M. and Dahia, P. L. M. (2023). Update on the genetics of paragangliomas. *Endocr Relat. Cancer* 30, e220373. 10.1530/ERC-22-0373 36748842 PMC10029328

[BIO060530C10] Goos, J. A. C. and Mathijssen, I. M. J. (2019). Genetic causes of craniosynostosis: an update. *Mol. Syndromol.* 10, 6-23. 10.1159/000492266 30976276 PMC6422124

[BIO060530C11] Huang, S., Song, J., He, C., Cai, X., Yuan, K., Mei, L. and Feng, Y. (2022). Genetic insights, disease mechanisms, and biological therapeutics for Waardenburg syndrome. *Gene Ther.* 29, 479-497. 10.1038/s41434-021-00240-2 33633356

[BIO060530C12] Kastriti, M. E., Faure, L., Von Ahsen, D., Bouderlique, T. G., Bostrom, J., Solovieva, T., Jackson, C., Bronner, M., Meijer, D., Hadjab, S. et al. (2022). Schwann cell precursors represent a neural crest-like state with biased multipotency. *EMBO J.* 41, e108780. 10.15252/embj.2021108780 35815410 PMC9434083

[BIO060530C13] Lackey, A. E. and Muzio, M. R. (2021). *DiGeorge Syndrome*. Treasure Island, FL: StatPearls. 31747205

[BIO060530C14] Lotfollahzadeh, S., Taherian, M. and Anand, S. (2021). *Hirschsprung Disease*. Treasure Island, FL: StatPearls. 32965813

[BIO060530C15] Marines, J., Lorenzini, F., Kissa, K. and Fontenille, L. (2023). Modelling 3D tumour microenvironment in vivo: a tool to predict cancer fate. *Curr. Issues Mol. Biol.* 45, 9076-9083. 10.3390/cimb45110569 37998746 PMC10670573

[BIO060530C16] Martik, M. L. and Bronner, M. E. (2021). Riding the crest to get a head: neural crest evolution in vertebrates. *Nat. Rev. Neurosci.* 22, 616-626. 10.1038/s41583-021-00503-2 34471282 PMC10168595

[BIO060530C17] Pla, P. and Monsoro-Burq, A. H. (2018). The neural border: Induction, specification and maturation of the territory that generates neural crest cells. *Dev. Biol.* 444 Suppl. 1, S36-S46. 10.1016/j.ydbio.2018.05.018 29852131

[BIO060530C18] Seal, S. and Monsoro-Burq, A. H. (2020). Insights into the early gene regulatory network controlling neural crest and placode fate choices at the neural border. *Front. Physiol.* 11, 608812. 10.3389/fphys.2020.608812 33324244 PMC7726110

[BIO060530C19] Simoes-Costa, M. and Bronner, M. E. (2013). Insights into neural crest development and evolution from genomic analysis. *Genome Res.* 23, 1069-1080. 10.1101/gr.157586.113 23817048 PMC3698500

[BIO060530C20] Simoes-Costa, M. and Bronner, M. E. (2015). Establishing neural crest identity: a gene regulatory recipe. *Development* 142, 242-257. 10.1242/dev.105445 25564621 PMC4302844

[BIO060530C21] Solovieva, T. and Bronner, M. (2021). Reprint of: schwann cell precursors: where they come from and where they go. *Cells Dev.* 168, 203729. 10.1016/j.cdev.2021.203729 34456178

[BIO060530C22] Tang, W. and Bronner, M. E. (2020). Neural crest lineage analysis: from past to future trajectory. *Development* 147, dev193193. 10.1242/dev.193193 33097550 PMC7595686

[BIO060530C23] Usman, N. and Sur, M. (2021). *CHARGE Syndrome*. Treasure Island, FL: StatPearls. 32644625

[BIO060530C24] Vega-Lopez, G. A., Cerrizuela, S., Tribulo, C. and Aybar, M. J. (2018). Neurocristopathies: new insights 150 years after the neural crest discovery. *Dev. Biol.* 444 Suppl. 1, S110-S143. 10.1016/j.ydbio.2018.05.013 29802835

[BIO060530C25] Wulf, A. M., Moreno, M. M., Paka, C., Rampasekova, A. and Liu, K. J. (2021). Defining pathological activities of ALK in neuroblastoma, a neural crest-derived cancer. *Int. J. Mol. Sci.* 22, 11718. 10.3390/ijms222111718 34769149 PMC8584162

